# Modification of the Physicochemical Properties of Active Pharmaceutical Ingredients via Lyophilization

**DOI:** 10.3390/pharmaceutics15112607

**Published:** 2023-11-09

**Authors:** Amir Taldaev, Denis I. Pankov, Roman P. Terekhov, Anastasia K. Zhevlakova, Irina A. Selivanova

**Affiliations:** 1 Institute of Biomedical Chemistry, 119121 Moscow, Russia; 2 Phystech School of Biological and Medical Physics, Moscow Institute of Physics and Technology, Institutskiy per. 9, 141701 Moscow, Russia; 3 Nelyubin Institute of Pharmacy, Sechenov First Moscow State Medical University, 119991 Moscow, Russia

**Keywords:** lyophilization, freeze drying, active pharmaceutical ingredients, modification of physicochemical properties, systematic review

## Abstract

Bioavailability is an important biopharmaceutical characteristic of active pharmaceutical ingredients (APIs) that is often correlated with their solubility in water. One of the methods of increasing solubility is freeze drying (lyophilization). The article provides a systematic review of studies published from 2012 to 2022 aimed at optimizing the properties of active pharmaceutical ingredients by freeze drying. This review was carried out in accordance with the recommendations of Preferred Reporting Items for Systematic Reviews and Meta-Analysis (PRISMA). In general, 141 modifications of 36 APIs attributed to 12 pharmacological groups were reported in selected publications. To characterize the products of phase modification after lyophilization, a complex of analytical methods was used, including microscopic, thermal, X-ray, and spectral approaches. Solubility and pharmacokinetic parameters were assessed. There is a tendency to increase solubility due to the amorphization of APIs during lyophilization. Thus, the alcohol lyophilizate of dihydroquercetin is “soluble” in water compared to the initial substance belonging to the category “very poorly soluble”. Based on the analysis of the literature, it can be argued that lyophilization is a promising method for optimizing the properties of APIs.

## 1. Introduction

Bioavailability is a term used to describe the percentage of an administered dose of a xenobiotic that reaches the systemic circulation [1]. It depends on the water solubility of active pharmaceutical ingredients (APIs) and their permeability through the intestinal wall. Orally administered drugs must have adequate systemic exposure to realize their pharmacological properties [2]. When oral bioavailability is low, plasma concentrations exhibit greater intersubjective variability. The portion of the substance that never reaches the systemic circulation is wasted, which may be considered an economic disadvantage for costly drugs [3]. The issue of increasing bioavailability is a current concern in pharmaceutical science.

The low bioavailability of APIs is often attributed to their limited solubility in water. Textbook methods to enhance solubility involve transforming poorly soluble substances into salt forms [4,5] or solid dispersions [6,7,8,9]. In addition to these common physicochemical methods, innovative approaches are used, such as co-crystallization [10,11,12], inclusion complexes [13,14,15,16], and reprecipitation from supercritical solvents [17,18]. Alongside these methods, water solubility can be optimized by lyophilization, the process of dehydration of the substance, which provides for pre-freezing of the solution and subsequent sublimation of ice in a vacuum. This technology was invented in 1890 and has been employed in the pharmaceutical industry since the 1950s [19].

Lyophilization offers a significant advantage compared to other modification methods. For instance, its absence of high-temperature exposure allows for its use with thermolabile APIs. Furthermore, the surface-to-volume ratio of the substance increases significantly after lyophilization, resulting in a greater specific surface area [18]. Additionally, the sublimated solvent can be reused [19]. Thus, freeze drying can be considered a “green” technology. The application of lyophilization involves the use of various solvents, solubilizers, and other excipients [20]. The product can be identified through spectral, X-ray, thermal, and morphological analysis. Phase modification impacts the solubility and pharmacokinetics of APIs, and there is a substantial body of data available. To our knowledge, a systematic review of API modification by lyophilization has not yet been conducted.

This study’s objective was to identify trends in the use of lyophilization for phase modifications in the properties of APIs.

## 2. Materials and Methods

### 2.1. Search Strategy

The systematic review was conducted following the Preferred Reporting Items for Systematic Reviews and Meta-Analyzes (PRISMA) guidelines [21]. The literature search covered the period from 2012 to 2022 and included earlier publications referenced in works from the past decade. The search strategy employed the keywords “lyophilization”, “pharmaceutical substance”, and “optimization” in combination with synonyms, signs, definitions, and logical functions. The search question was formulated as: (“freeze drying” OR “lyophilization”) AND (“pharmaceutical substances” OR “active pharmaceutical ingredient”) AND (modification OR optimization) AND -review AND (“comparative analysis” OR comparison).

### 2.2. Review Protocol and Data Extraction

**Figure 1:**
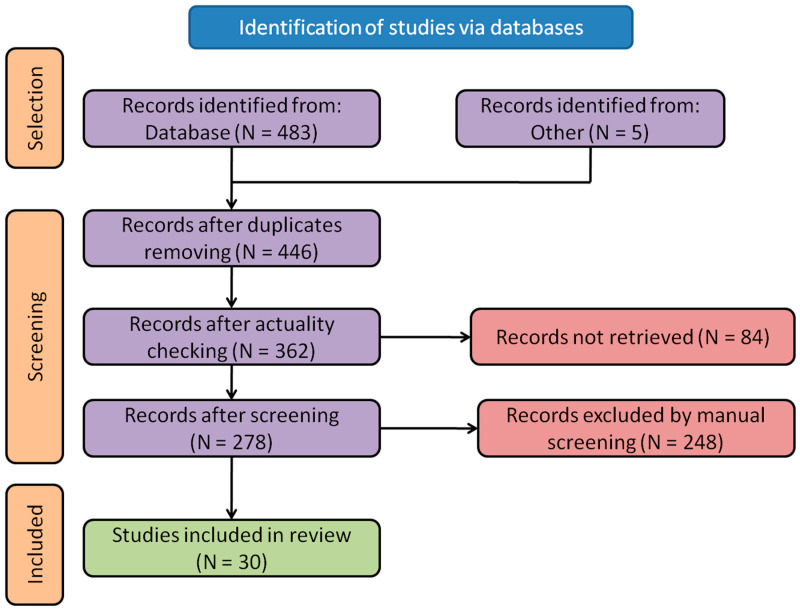
PRISMA flowchart of the search and selection process for the articles.

Two independent review authors (D.P. and A.T.) conducted the literature search in the Google Scholar database. The results of the search were collected on Google Drive. The same review authors independently screened publications using the criteria for inclusion and exclusion (Table 1):

In cases of disagreement, decisions were reached through discussion and consultation with three other authors (R.T., A.Z., and I.S.). As a result, 30 articles underwent a full-text review (Figure 1). Qualitative and quantitative content analysis, as well as synthesis and necessary statistical analyses, were performed by D.P. and A.T. This study outcomes were presented through narrative synthesis, tables, and figures.

### 2.3. Assessment of Risk of Bias

Two authors (R.T. and A.Z.) systematically and independently assessed the risk of bias in each paper reporting about the improvements in APIs solubility after lyophilization. This analysis considered two bias domains: the proof of phase transition and the validation of the quantitative analytical method used in solubility tests. For the first domain, a low risk of bias was determined when the article reported at least two methods, with one of them being X-ray powder diffraction (XRPD). Other methods for analyzing the phase structure included scanning electron microscopy (SEM), differential scanning calorimetry (DSC), and Fourier transform infrared spectroscopy (FTIR). If only one approach was used or XRPD data were absent, the risk of bias was considered high. The second domain focused on the validation of the analytical assay used in solubility testing, assessing specificity, limit of quantitation, linearity, trueness, precision, analytical range, and robustness. The term “specificity” is defined as the ability of an analytical method to distinguish the analyte from other chemicals in the sample. For example, in HPLC methods, specificity can be assessed through the resolution value between the major peak and the nearest ones. It should be more than 1.5. The limit of quantitation represents the lowest concentration of the analyte in a sample that can be determined with acceptable precision and accuracy. Precision is evaluated by calculating the relative standard deviation, or coefficient of variance. A low risk of bias was assigned when the relative standard deviation was less than or equal to 2.0%. Trueness was confirmed by examining the deviation from the label claim or by comparing results with those obtained from a validated or reference method. The analytical range required acceptable levels of trueness, precision, and a linear relationship between concentration and peak area. Linearity was assessed through the construction of calibration curves, with a correlation coefficient equal to or greater than 0.99 indicating a low risk of bias. Robustness was evaluated based on the method’s ability to maintain parameters (pH value, mobile phase composition, column specifications, temperature, and flow rate) within slight variations.

When articles lacked information on these issues, the risk of bias was deemed unclear. Disagreements in bias assessments were resolved through discussion or, when necessary, by an independent third review author (I.S.). The results of bias assessments were presented in a risk of bias graph and narrative synthesis.

## 3. Results

### 3.1. General Outcomes

**Figure 2:**
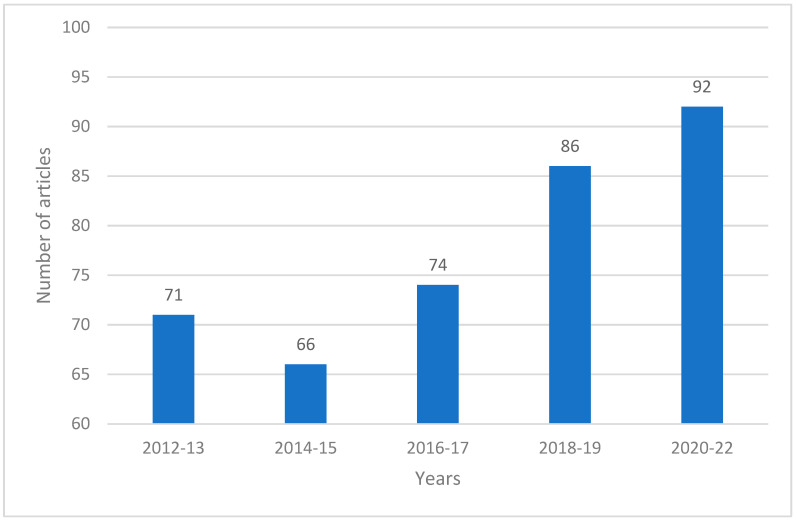
Monitoring of scientific information on physicochemical modification of API via lyophilization (Google Scholar data).

A gradual increase in the number of articles on a given topic per year was observed during this study period (Figure 2).

We found that the modification of API properties through lyophilization was the focus of research from Russia, Romania, Egypt, Brazil, Germany, Turkey, Malaysia, and the USA. However, the majority of articles were authored by researchers affiliated with scientific institutions in Japan and India.

**Figure 3:**
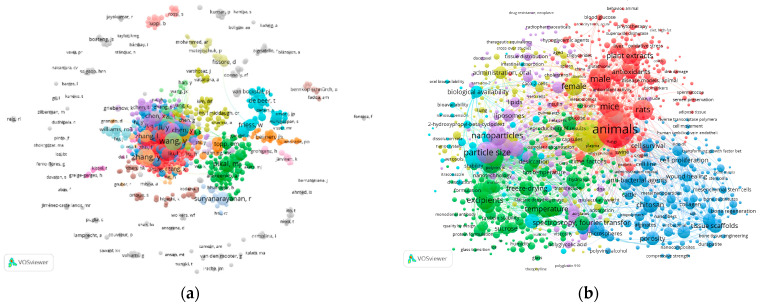
Bibliometric networks on (**a**) co-authorship relations, (**b**) co-occurrence relations. Search question: (“freeze drying” OR “lyophilization”) AND pharma*.

During the analysis of researcher groups, we found 91 clusters of co-authors (Figure 3a). The largest one includes more than 180 co-authors. Big clusters are active nowadays and are continuing the data generation.

In addition, we analyzed the co-occurrence relations using the author’s key words and MeSH terms (Figure 3b). The outcomes demonstrate the prevalence of preclinical studies and clinical trials in the current literature. Also, technological tasks and excipients are the focus of investigators.

### 3.2. APIs as an Objects of Lyophilization

In general, 141 modifications of 36 APIs, spanning 12 pharmacological groups, were reported in the selected publications (Table 2).

The largest number of modified congeners were found among non-narcotic analgesics, antifungals, and hypotensive remedies. Antimigrenous, antipsychotic, and antiemetic drugs were represented by naratriptan, quetiapine, and domperidone, respectively. Additionally, the antifungal pharmacological group had the highest number of phase modifications and the fewest number of antimigrenous drugs. However, among APIs, docetaxel, an antitumor remedy, had the greatest number of modifications achieved through lyophilization. It is worth noting that not all of the modified congeners are utilized in clinical practice. For instance, 1-(4-nitrophenyl)-3-(2,2,4,4-tetramethylthiochroman-6-yl)thiourea (SHetA2) is in Phase 1 of clinical research, and benzyl 4-(2-hydroxy-5-nitrophenyl)-2,6,6-trimethyl-5-oxo-1,4,5,6,7,8-hexahydroquinoline-3-carboxylate (M3) is also not in common clinical use.

### 3.3. Excipients in Lyophilization

During the development of lyophilized formulations, various technological challenges emerged, which were successfully addressed through the incorporation of excipients (Table 3). Water and ethanol were the most commonly used solvents, while polysorbate and polyethylene glycol were frequently employed as solubilizers. Cryoprotectants also played a significant role as excipients, frequently used in the lyophilization process.

For lyophilizates, the resulting dosage from research encompasses nanosuspensions [47], powders for inhalation [53], and orally disintegrating tablets [31].

### 3.4. Methods of Lyophilizate Analysis

To characterize the products resulting from the phase modification through lyophilization, a comprehensive set of analytical methods was employed, including microscopic, thermal, X-ray, and spectral approaches (Table 4). Most substances were analyzed using XRPD and DSC. SEM emerged as the preferred method for describing substance morphology. Spectral methods, such as FTIR and spectroscopy of nuclear magnetic resonance (NMR), were used less frequently than other techniques.

**Figure 4:**
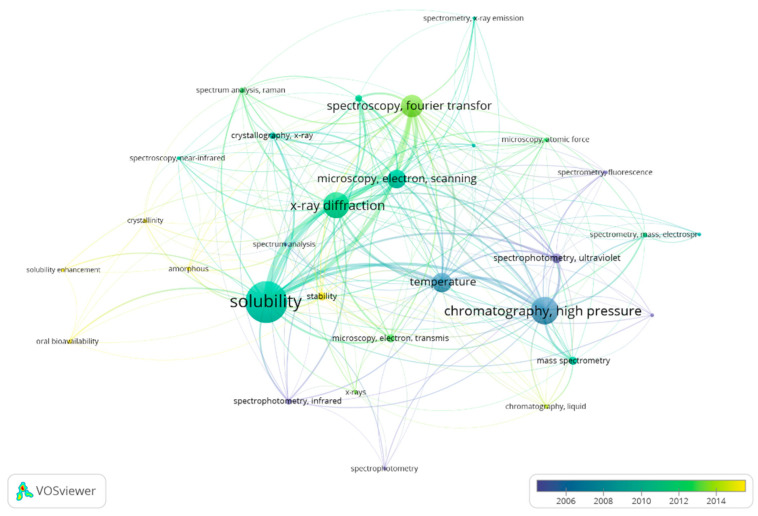
Bibliometric networks on methods of lyophilizate analysis.

The following analysis of literature data showed that solubility and phase state were often the focus of researchers (Figure 4). HPLC was used frequently in the analysis of lyophilizates until 2008. However, nowadays, investigators pay more and more attention to the stability of freeze-dried products. The number of articles that focus on oral bioavailability and solubility enhancement is not so high at the moment. Nevertheless, the majority of papers on these topics were published recently, so there is a trend toward them.

### 3.5. Influence of Lyophilization on Morphology and Physicochemical Properties

**Figure 5:**
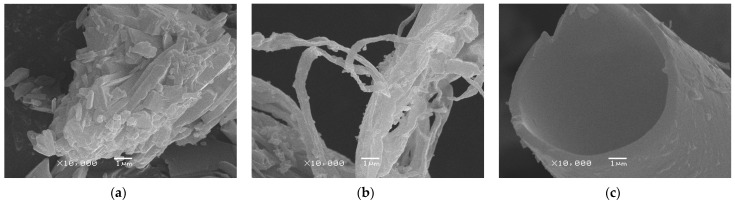
Photomicrography of different dihydroquercetin forms: (**a**) dihydroquercetin powder before lyophilization at 10,000 magnification; (**b**) lyophilizate from ethanol at 10,000 magnification; (**c**) lyophilizate from acetonitrile at 10,000 magnification [44].

The lyophilizates differ in morphology. For example, before lyophilization, dihydroquercetin was a fine powder, and the morphology of its units can be characterized as agglomerates (Figure 5a). After lyophilization of ethanol and acetonitrile in aqueous solutions of dihydroquercetin, the morphology turned to fibers (Figure 5b) and vessels (Figure 5c), respectively. The size of particles decreased from 1.6 to 86.4 times [44].

It is important to notice the different physicochemical properties of lyophilizates. For instance, initial substances and their lyophilizates differ in water solubility (Table 5). Miconazole exhibited the lowest water solubility at 0.004 µg/mL, which increased significantly to 548 µg/mL after lyophilization with tartaric acid. Conversely, a mixture of meloxicam and paracetamol demonstrated the highest initial solubility (5190 µg/mL), which further improved to 37,730 µg/mL post-lyophilization. The application of lyophilization consistently optimized water solubility for all APIs, though the degree of improvement varied among different cases. For example, lyophilization increased the solubility of gliclazide by 1.1-fold, while quench cooling achieved a 2.2-fold increase. The extent of solubility enhancement ranged from 1.1 to an impressive 137,150 times, contingent on the type of freeze drying and the excipients used. Notably, the presence of succinic acid led to a 97.5-fold increase in the solubility of miconazole, though it was less effective compared to slurry. In contrast, when tartaric acid was introduced, lyophilization emerged as the superior approach for achieving the goals of phase modification.

### 3.6. Influence of Lyophilization on Pharmacokinetic

Pharmacokinetic parameters were evaluated for seven modifications of APIs (Table 6). In all reported cases, lyophilization led to an increase in maximum plasma concentration (C_max_) and area under the curve (AUC). The most pronounced AUC increase was observed for tranilast and nobiletin, with increments of 18.3 and 17.8, respectively. However, the time until the maximum plasma concentration (T_max_) exhibited varying changes among different modifications. For tranilast lyophilizate, it is decreased by a factor of 3.3 times, whereas the lyophilizate of miconazole with tartaric acid increases this parameter by a factor of 3.1.

### 3.7. Risk of Bias

Possible forms of bias were assessed for 17 articles from Table 5, following the methodological approach designed for this systematic review.

In 14 papers, the phase state of samples before and after lyophilization was analyzed using a combination of methods, including SEM, DSC, and XRPD. Thus, the risk of bias in providing evidence of phase transition scans is considered acceptable for the majority of articles. However, the XRPD data for ellagic acid nanosponges were not available.

The assessment also included parameters such as precision, limit of quantitation, and trueness (accuracy), which were addressed in all articles. Specificity was evaluated in methods for determining quetiapine and meloxicam-paracetamol. The analytical range was defined in all cases, except for the methods concerning meloxicam and paracetamol. The limit of detection was calculated in all studies, except for the docetaxel methods. The resolution between the peak of the analyzed compound and its nearest neighbor ranged from 1.98 to 6.00, while quantitation limits varied from 0.000043 to 0.3 µg/mL. The correlation coefficient between the amount of the analyte in the sample and the peak area within the analytical range exceeded 0.998. The relative standard deviation ranged from 0.24 to 6.03%, with the coefficient of variance between 0.215 and 0.483%. The percent relative error ranged from 0.15 to 8.14%. The standard error values were 0.11 and 0.36. To evaluate the bioavailability of the koumine inclusion complex with cyclodextrin, the UPLC/MS method was validated, although researchers did not provide validation for the assay API in the solubility evaluation method. For assessing the solubility of the binary solid dispersion of meloxicam and paracetamol, a validated HPLC method was used to quantitatively evaluate lornoxicam and paracetamol.

**Figure 6:**
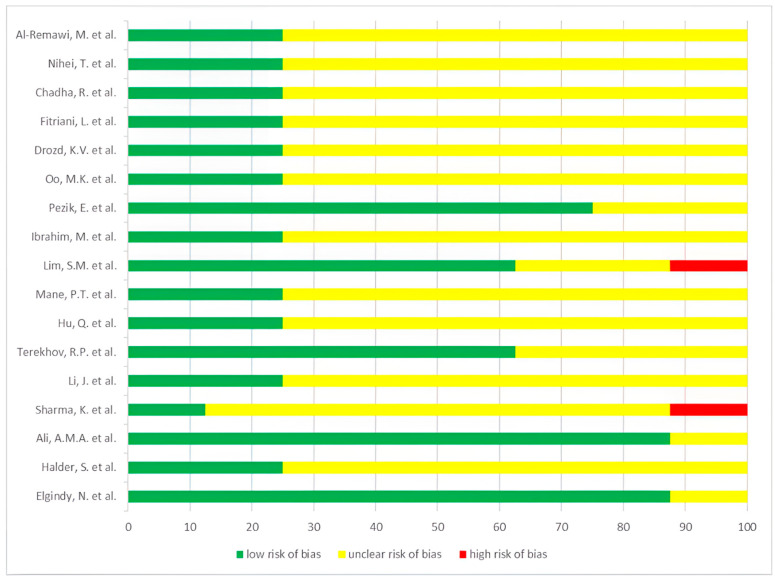
Risk of a bias graph [49,50,43,30,29,48,47,40,36,39,38,32,34,33,35,26,37].

A summary of the low, high, or unclear risk for bias assessment of the analyzed articles is presented in Figure 6.

The articles [32,35,36,47] can be characterized by a set of parameters with a low risk of bias.

## 4. Discussion

This systematic review aimed to consolidate and analyze information related to the use of lyophilization as a method for phase modification of APIs. The observed increase in the number of articles on this topic underscores its relevance in pharmaceutical science. This trend can be attributed to the high costs, time, and expertise required for drug discovery. Prompting researchers to explore the potential of well-known compounds and investigating API phase modifications can be seen as a pragmatic compromise. The adoption of intellectual technologies has accelerated the development of pharmaceuticals [54]. The combination of expertise in solid-state chemistry and drug delivery has piqued the interest of scientists, particularly from Japan and India, in the field of lyophilization.

The current investigation has found that the majority of selected APIs for phase modification are characterized by low water solubility and high permeability. Therefore, they can be classified as Class II in the Biopharmaceutical Classification System [55]. Surprisingly, among the analyzed papers, there is one dedicated to a new substance that is in the clinical study stage. This observation indicates that optimizing phase states is becoming a new stage in rational drug development. Regarding excipients, researchers prefer to use non-toxic and low-toxic solvents that align with the principles of “green” chemistry [56,57]. Dissolution is a critical stage in lyophilization, and different solubilizers are frequently used. To prevent the destructive effects of low temperature and pressure, sugar alcohols are added before the lyophilization process, serving as cryoprotectants [58].

In recent years, there has been a trend toward using lyophilization in combination with other technological approaches (14% of articles). For instance, budesonide suspension was obtained by dissolving it in supercritical carbon dioxide and then lyophilizing it [22]. A mixture of meloxicam and paracetamol underwent ultrasound treatment before [49] freezing. Gliclazide solution was lyophilized and homogenized under high pressure [43]. In general, tandem methods enhance the efficiency of API property modification. However, it is worth noting that in the majority of the articles found, the relationship between lyophilization conditions and product properties was not studied.

Turning to the analytical methods used in selected papers, researchers pay significant attention to the crystallinity of products, which can be studied using DSC or XRPD. In several articles, both methods are employed to prevent biases associated with using a single approach [59]. Single-crystal systems exhibit greater thermodynamic stability [60]. This product can be obtained via lyophilization of suspensions, forming amorphous solids from solutions. However, spectral methods are less frequently utilized. Additionally, we did not find any Raman spectroscopy data, which may create a knowledge gap in the chemical understanding of new solid formation.

The vast majority of papers describe improvements in the solubility profiles of substances, with the best solubility observed in amorphous forms. These observed changes in physicochemical properties are associated with an increase in the surface area of API particles [61]. Another explanation for better solubility is the “spring and parachute” effect, commonly seen in crystal engineering [62,63]. Oo et al. discovered that better solubility is associated with an increase in the permeability of lyophilizate [48]. The passage through the cell membrane of raw and lyophilized nisoldipine was 31.26% and 51.55%, respectively [48].

Our systematic review focuses on researching biopharmaceutical and pharmacological parameters. To simulate natural drug release conditions, solubility tests are often conducted in buffer solutions or media that simulate biological fluids rather than in water [27]. Since 2017, there has been an increased emphasis on evaluating the pharmacodynamics and pharmacokinetics of modified forms in vivo [64]. Apparently, the improved pharmacokinetic parameters are associated with increased water solubility.

To assess the reliability of the findings in our current systematic review, it was crucial to conduct a bias risk analysis of the included papers. Guidelines for considering bias in papers for randomized controlled clinical trials and preclinical studies exist [65,66]. Previously, we proposed a similar tool for in silico studies [54], which has been applied in several systematic reviews [67,68]. To the best of our knowledge, no such approach exists for studies in the field of pharmaceutical analysis. Therefore, we have suggested criteria based on specifications, scientific literature, and discussions with professional society to serve as references. The domains we have included cover various types of bias that may affect the interpretation of the analysis results.

XRPD is considered the “golden standard” for solid state analysis and is widely used in pharmaceutical chemistry to control API polymorphism [69]. However, several articles have reported that relying solely on one method for identifying the solid phase may result in irrelevant results [59,70,71]. Validation requirements are derived from the State Pharmacopoeia of the Russian Federation, which is harmonized with the European Pharmacopeia [72,73].

After assessing the risk of bias in selected articles, we found that there is no high risk of bias in these studies. While all articles meet the requirements for proving phase transitions, some articles did not report the validation of quantitative analysis in the solubility test. The absence of validation parameters affects the relevance and applicability of analytical results to pharmaceutical science. Nevertheless, this trend emphasizes the need to pay more attention to the quality of such papers. However, we did not identify any conflicts of interest in the analyzed studies.

The strength of our present study lies in its inclusion of articles published in peer-reviewed journals. The size of the dataset is substantial, and the number of observed API modifications is extensive. Nonetheless, this systematic review has some limitations. The included studies exhibit heterogeneity in the nature of analyzed compounds, the methods of generation and analysis, and the reporting of pharmacological data alongside solubility profiles. We could not assess publication bias using a Begg funnel plot or an Egger test due to the heterogeneity of the included studies in terms of the APIs analyzed. In general, we may have missed potentially eligible studies published in languages other than English or Russian, as well as studies with negative results.

To summarize, our findings reveal relevant trends and provide direction for future studies on lyophilization as a method of phase modification. This work underscores the need to delve deeper into the correlation between lyophilization conditions and product properties, as well as the chemical fundamentals of observed phase transitions. Accumulating pharmacological and biopharmaceutical data will enable us to generalize these findings in meta-analyses.

## 5. Conclusions

This systematic review delves into the trends surrounding the use of lyophilization for targeted modifications in the properties of Active Pharmaceutical Ingredients (APIs). Building upon these insights, it becomes evident that lyophilization holds great promise as a versatile approach for tailoring the properties of Active Pharmaceutical Ingredients (APIs). The capacity of lyophilization to optimize the physicochemical and biopharmaceutical characteristics of APIs underscores its significance in pharmaceutical research and development. However, it is crucial to address the recurrent issue of validation in quantitative analysis, which hampers the reliability and credibility of research findings. Researchers should prioritize robust validation protocols to ensure that their studies meet the rigorous standards of scientific inquiry. This step is essential to maintaining the integrity of the field and enhancing the trustworthiness of research outcomes. Furthermore, the absence of spectral data for products resulting from lyophilization-induced phase modifications are a notable gap. Bridging this void could provide a deeper chemical understanding of the processes involved, potentially unlocking new avenues for innovation in drug development. Incorporating spectral analysis into research methodologies may shed light on the phase transformations occurring during lyophilization. In spite of this study’s inherent limitations, it undeniably enriches our comprehension of the current state of the field. Specialists in drug discovery can draw valuable insights from this review, guiding them in making informed decisions and advancing their research endeavors. As a result, the field can pave the way for more effective drug development practices and, ultimately, better patient outcomes.

## Figures and Tables

**Table 1:** Overall selection criteria for publication screening.

Section	Inclusion Criteria	Exclusion Criteria
Language of full-text	English	Any other language
	Russian	
Publication type	Experimental studies (in silico, in vitro, and in vivo)	Reviews
		Editorials
		Letter to the editor
Content	Use of lyophilization for physicochemical modification of API	Use of lyophilization for any other purposes then physicochemical modification of API
		Use of lyophilization for physicochemical modification of inactive ingredients
Access	Full-text article evaluable	Abstract only evaluable

**Table 2:** Congeners, that were modified by lyophilization.

Pharmacological Group	Congeners	Number of Modifications	Source
Antiallergic	Budesonide	1	[22]
	Tranilast	8	[23]
Antibiotics	Ampicillin	1	[24]
	Fusidic acid	3	[25]
Antiemetic	Domperidone	5	[26]
Antifungal	Fluconazole	3	[27]
	Itraconazole	3	[27]
	Ketoconazole	6	[28]
	Miconazole	9	[29]
	Usnic acid	2	[30]
Antimigrenous	Naratriptan	2	[31]
Antioxidant	Dihydroquercetin	6	[32]
	Ellagic acid	4	[33]
	Naringin	4	[34]
	Neohesperidin	4	
Antipsychotic	Quetiapine	6	[35]
Antitumor	Docetaxel	13	[36]
	Flutamide	9	[37]
	Koumine	1	[38]
	Lapatinib	3	[39]
	SHetA2	2	[40]
Antiviral	Efavirenz	10	[41]
	Saquinavir	3	[42]
Hypoglycemic	Gliclazide	3	[43]
	Glipizide	2	
	Repaglinide	2	
Hypotensive	Carvedilol	2	[44]
	Enalapril	1	[45]
	Indapamide	1	[46]
	M3	7	[47]
	Nifedipine	6	[28]
	Nisoldipine	3	[48]
Non-narcotic analgesics, including nonsteroidal anti-inflammatory drugs	Meloxicam	7	[49]
	Naproxen	6	[31]
	Nobiletin	1	[50]
	Paracetamol	7	[49]
	Tiaprofenic acid	1	[51]

**Table 3:** Excipients that were used in the phase modification of APIs.

Group	Excipient	Number of Modifications	Source
Solvents	Ethanol	37	[36]
	Aceton	1	[49]
	Water	83	[25]
	Methanol	8	[29]
	CO_2_	6	[22]
	1,4-dioxane	10	[23]
	Acetonitrile	3	[32]
	Tetrahydrofuran	10	[52]
	Tertiary butyl alcohol	12	[37]
Solubilizers	Cyclodextrin	9	[45]
	Polyvinylpyrrolidone	8	[48]
	Poloxamer	5	[48]
	Kolliphor^®^	8	[40]
	Hydroxypropylcellulose	9	[23]
	Hydroxypropylmethylcellulose	6	[23]
	Eudragit^®^	1	[23]
	Sodium lauryl sulfat	7	[41]
	Polysorbate	10	[52]
	Polyethylenglicol	13	[37]
	Kollidon 12PF	6	[36]
	SoluPlus^®^	13	[36]
	Lutrol F 68	3	[36]
Lubricants	Dipalmitoylphosphatidylcholine	1	[22]
	Monoglyceride of hydrogenated palm oil	1	[22]
	Monoglyceride laurate	1	[22]
	Monoglyceride palmitate	1	[22]
	Monoglyceride stearate	1	[22]
	Sorbitanmonopalmitate	1	[22]
Cryoprotectants	Lactin	3	[52]
	Mannitol	3	[52]
	Sucrose	4	[52]
	Trehalose	3	[40]

**Table 4:** Methods of lyophilizates characterization.

API	Morphological Analysis		Thermal Analysis			X-ray Analysis		Spectral Analysis		Source
	SEM *	PLM *	HSM *	DSC *	TGA *	XRPD *	XRSC *	FTIR *	NMR *	
Ampicillin				+						[24]
Budesonide	+			+		+				[22]
Dihydroquercetin	+			+	+	+		+		[32]
Docetaxel	+			+		+		+		[36]
Domperidone	+	+		+		+		+		[26]
Efavirenz	+			+	+	+				[41]
Ellagic acid	+							+		[33]
Enalapril				+		+		+		[45]
Flutamide				+		+				[37]
Fusidic acid	+				+	+		+		[25]
Indapamide	+			+	+	+		+	+	[46]
Koumine	+			+		+		+	+	[38]
Lapatinib	+			+		+		+		[39]
M3				+		+		+		[47]
Meloxicam, paracetamol	+			+		+		+		[49]
Miconazole	+			+		+				[29]
Naproxen, naratriptan	+			+						[31]
Neohesperidine, naringin	+			+		+		+		[34]
Nifedipine, ketoconazole				+		+		+		[28]
Nisoldipine						+		+		[48]
Nobiletin	+			+		+				[50]
Quetiapine	+			+		+		+		[35]
Repaglinide, gliclazide, glipizide				+		+				[43]
SHetA2				+		+				[40]
Tiaprofenic acid	+			+		+				[51]
Tranilast	+	+		+		+		+		[23]
Usnic acid	+			+		+		+		[30]

* SEM—scanning electron microscopy; PLM—polarized light microscopy; HSM—hot stage microscopy; DSC—differential scanning calorimetry; TGA—thermogravimetric analysis; XRPD—X-ray powder diffraction; XRSC—X-ray single crystal; FTIR—Fourier transform infrared spectroscopy; NMR—nuclear magnetic resonance.

**Table 5:** The solubility of raw APIs and these lyophilizates.

API	Excipients	Modification Method	Solubility (µg/mL)		Increase of Solubility	Source
			Before	After		
Miconazole	Succinic acid	Liquid-Assisted Grinding	0.004	0.80	×200.0	[29]
		Slurry		0.89	×222.5	
		Lyophilization		0.39	×97.5	
	Maleic acid	Liquid-Assisted Grinding		3.82	×955.0	
		Slurry		4.30	×1075.0	
		Lyophilization		13.43	×3357.5	
	Tartaric acid	Liquid-Assisted Grinding		17.45	×4362.5	
		Slurry		85.26	×21,315.0	
		Lyophilization		548.60	×137,150.0	
SHetA2	Kolliphor^®^ HS 15, trehalose	Ultra rapid lyophilization	0.020	10.26	×513.0	[40]
		Spray lyophilization		8.14	×407.0	
Domperidone	SoluPlus^®^+ Kolliphor^®^ P 188	Lyophilization	0.470	27.54	×58.6	[26]
		Vacuum evaporation		12.97	×27.6	
Quetiapine	Nicotinamide	Lyophilization	1.710	25.01	×14.6	[35]
Nobiletin	Hydroxypropylcellulose	Lyophilization	7.500	33.00	×4.4	[50]
Docetaxel	Hydroxypropyl-β-cyclodextrin	Lyophilization	8.210	4.71	×0.6	[36]
	SoluPlus^®^			231.84	×28.2	
	Kollidon 12PF			9.95	×1.2	
	Kollidon 12PF + Lutrol F 68			14.90	×1.8	
	Hydroxypropyl-β-cyclodextrin + SoluPlus^®^			238.68	×29.1	
Flutamide	Poloxamer 188	Lyophilization	13.000	35.00	×2.7	[37]
	Polyethylenglicol		13.000	34.00	×2.6	
	Polyvinylpyrrolidone		12.000	26.00	×2.2	
Lapatinib	β-cyclodextrin	Lyophilization	20.210	27.94	×1.4	[39]
		Kneading		25.46	×1.3	
	β-cyclodextrin + Polyvinylpyrrolidone	Lyophilization		57.97	×2.9	
Gliclazide	-	Lyophilization	56.300	61.80	×1.1	[43]
		Quench cooling		121.20	×2.2	
		Vacuum evaporation		68.00	×1.2	
Neohesperidin	-	Lyophilization	61.000	1550.00	×25.4	[34]
	Naringin		-	4870.00	-	
Nisoldipine	Polyvinylpyrrolidone, poloxamer	Lyophilization	63.330	123.89	×2.0	[48]
		Vacuum evaporation		111.85	×1.8	
		Hot melt mixing		117.41	×1.9	
M3	Poloxamer 188, trehalose	Precipitation + ultrasonication+ lyophilization	78.800	203.30	×2.6	[47]
Ellagic acid	Cyclodextrin	Melting + Lyophilization	162.500	721.00	×4.4	[33]
		Sonification + Lyophilization		607.00	×3.7	
Usnic acid	Hydroxypropylmethylcellulose	Lyophilization	227.000	932.00	×4.1	[30]
		Spray drying		576.00	×2.5	
Dihydroquercetin	Ethanol	Lyophilization	700.000	3090.00	×4.4	[32]
	Acetonitril			2140.00	×3.1	
Koumine	Hydroxypropyl-β-cyclodextrin	Lyophilization	700.000	1810.00	×2.3	[38]
Meloxicam+paracetamol	-	Lyophilization with sonification	5190.000	37,730.00	×7.3	[49]
		Hot evaporation		12,300.00	×2.4	

**Table 6:** Pharmacokinetic parameters of raw APIs and these lyophilizates.

API	Sample	C_max_ (µg/mL)	T_max_ (h)	AUC_0-inf_ (µg•h/mL)	Source
Efavirenz	Raw	0.600	3.00	2.600	[41]
	Lyophilizate	1.300	1.50	9.800	
Koumine	Raw	0.023	0.33	0.030	[38]
	Lyophilizate	0.050	0.30	0.078	
Miconazole	Raw	0.120	2.00	1.372	[29]
	Miconazole+succinic acid	0.261	1.30	3.296	
	Miconazole+maleic acid	0.365	2.20	4.017	
	Miconazole+tartaric acid	0.379	6.20	6.349	
Nobiletin	Raw	0.087	3.00	0.230	[50]
	Lyophilizate	1.200	1.20	4.100	
Tranilast	Raw	0.100	1.80	0.800	[23]
	Lyophilizate	4.600	0.54	14.600	

## Data Availability

Data are contained within the article.
